# Advancing viral RNA structure prediction: measuring the thermodynamics of pyrimidine-rich internal loops

**DOI:** 10.1261/rna.059865.116

**Published:** 2017-05

**Authors:** Andy Phan, Katherine Mailey, Jessica Saeki, Xiaobo Gu, Susan J. Schroeder

**Affiliations:** 1 Department of Chemistry and Biochemistry; 2 Department of Microbiology and Plant Biology, University of Oklahoma, Norman, Oklahoma 73019, USA

**Keywords:** internal ribosome entry site, viral RNA, thermodynamics, pyrimidine-rich internal loops, RNA secondary structure prediction

## Abstract

Accurate thermodynamic parameters improve RNA structure predictions and thus accelerate understanding of RNA function and the identification of RNA drug binding sites. Many viral RNA structures, such as internal ribosome entry sites, have internal loops and bulges that are potential drug target sites. Current models used to predict internal loops are biased toward small, symmetric purine loops, and thus poorly predict asymmetric, pyrimidine-rich loops with >6 nucleotides (nt) that occur frequently in viral RNA. This article presents new thermodynamic data for 40 pyrimidine loops, many of which can form UU or protonated CC base pairs. Uracil and protonated cytosine base pairs stabilize asymmetric internal loops. Accurate prediction rules are presented that account for all thermodynamic measurements of RNA asymmetric internal loops. New loop initiation terms for loops with >6 nt are presented that do not follow previous assumptions that increasing asymmetry destabilizes loops. Since the last 2004 update, 126 new loops with asymmetry or sizes greater than 2 × 2 have been measured. These new measurements significantly deepen and diversify the thermodynamic database for RNA. These results will help better predict internal loops that are larger, pyrimidine-rich, and occur within viral structures such as internal ribosome entry sites.

## INTRODUCTION

Amidst a flood of sequence information, lightening-fast development of RNA therapeutics, whirlwind discoveries of novel RNA gene regulation functions, and the thunderous threat of outbreaks from viral RNA pathogens such as Ebola and Zika, RNA thermodynamics provide a compass to navigate the storm. Accurate thermodynamic measurements of RNA motifs form the core for a wide array of approaches to predicting RNA structure and function from sequence (for example, Xu et al. 2014; Eggenhofer et al. 2016; Xu and Mathews 2016). These prediction tools are a critical resource for responding to the deluge of sequence information generated by ever-advancing technology. For example, a conference on the challenges of RNA structure prediction lists as the top priority improvements in thermodynamics measurements: “Nearest-neighbor interactions are robust parameters that represent a central anchor for all of secondary structure prediction. However, the context dependence of these terms and their conditional variation make it important to continually improve them in order to advance the power of prediction algorithms” (Pyle and Schlick 2016). Accurate thermodynamic parameters also enable better three-dimensional structure prediction. For example, only the prediction tool that included the most updated thermodynamic parameters for GU pairs accurately predicted the structure of four consecutive terminal GU pairs (Gu et al. 2015). Thus, we present 40 new thermodynamic parameters for asymmetric loop motifs that are underrepresented in the thermodynamic database but frequently occur in viral RNA. We also present an updated database and prediction rules for all 194 free energy measurements of RNA asymmetric internal loops. The database of thermodynamic parameters is a foundation upon which prediction algorithms build to provide a launch pad for hypotheses on novel noncoding RNA function, screening of viral RNA drug target sites, and design of RNA therapeutics.

Although rigorous measurement of the thermodynamic stabilities for all possible internal loop sequences is not realistic, a diverse and deep sampling of possible sequences can provide a foundation for estimating the thermodynamic stabilities of loop sequences and thus aid RNA structure prediction tools. Accurately predicting secondary structure from sequence is an integral step in understanding RNA structure and function. Internal loops occur frequently within RNA structure and often form part of enzyme active sites, protein interactions, metal ion binding sites, or drug targets. The current model used to predict internal loops and RNA secondary structure was last updated in 2004 and has an accuracy of ∼76% when compared to known RNA structures that have been determined through chemical modification or phylogenetic analysis (Mathews et al. 2004). This model predicts well small loops of <6 nt, but inaccuracies increase with loops of larger sizes that had not yet been studied in the 2004 database. The 2004 thermodynamic database is biased toward smaller, purine-rich internal loops. Symmetric, 2 × 2 loops (a loop that has 2 nucleotides [nt] opposite 2 nt) are overrepresented in the thermodynamic database, whereas loops that are pyrimidine-rich or larger than size 3 × 3 are underrepresented. Opposite trends are observed in loops that naturally occur in internal ribosome entry sites (IRES) (Mokrejs et al. 2010). The 2004 model surveyed RNA secondary structures from different species of bacteria, yeast, and mammals, but did not include any viral RNA structures (Mathews et al. 2004). Thus, new viral RNA structures and new measurements on more diverse loops can improve RNA structure prediction tools.

Many pathogenic viruses such as HIV, polio, foot and mouth, and hepatitis use internal ribosome entry sites (IRES) to produce viral proteins (Plank and Kieft 2012; Lozano and Martinez-Salas 2015). Internal loops and bulges that occur within the Hepatitis C IRES have been extensively studied as known drug target sites (Kikuchi et al. 2005; Davis and Seth 2011; Dibrov et al. 2014). In vitro studies and crystal structures demonstrate that small molecules such as benzimidazole bind to the loop motifs within the HCV IRES, induce a conformational change, and effectively inhibit viral translation (Paulsen et al. 2010; Dibrov et al. 2012). Current thermodynamic parameters poorly predict these loop motifs within viral IRESs, and thus more accurate RNA structure predictions can guide better design of viral therapeutics and selection of drug target sites.

The differences between the thermodynamic database and the IRES database may be a contributing factor in the inaccuracies of the current prediction model (Mathews et al. 2004; Mokrejs et al. 2010). This article presents new thermodynamic parameters for 40 loops and significantly diversifies the thermodynamic database. The NMR data show that pyrimidine-rich loops are stabilized by multiple UU pairs. A revised model for the prediction of asymmetric loops is proposed that includes bonus terms for UU pairs in the middle of loops and protonated CC pairs. In addition, new initiation terms for loops consisting of 6, 7, and 8 nt are presented from analysis of an updated database of 507 internal loop measurements.

## RESULTS AND DISCUSSION

### Database analysis

**FIGURE 1.**
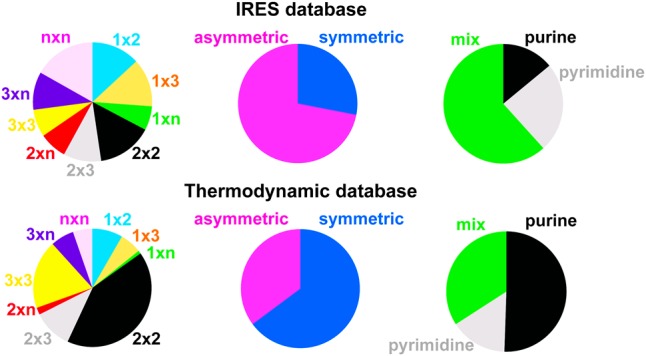
A comparison of the internal loop characteristics in viral IRES and the thermodynamic database for RNA structure prediction. Data for RNA internal loops are from an IRES database (Mokrejs et al. 2010) (*top* row). The RNA secondary structures in the IRES database were experimentally determined from phylogeny and chemical or enzymatic probing. Analysis for RNA internal loops in the thermodynamic database (Santa Lucia et al. 1990, 1991; Peritz et al. 1991; Walter et al. 1994; Wu et al. 1995; Schroeder et al. 1996; Xia et al. 1997; Schroeder and Turner 2000, 2001; Burkard et al. 2001; Chen et al. 2004, 2005, 2006, 2009; Bourdelat-Parks and Wartell 2005; Chen and Turner 2006; Badhwar et al. 2007; Christiansen and Znosko 2008, 2009; Hausmann and Znosko 2012) (*bottom* row). Loop sizes are described by the number of nucleotides on each side of a loop. For example, a 1 × 3 loop has 1 nt opposite 3 nt. *N* is any number >3 nt. When the number of nucleotides on each side of the loop is different, then the loop is described as asymmetric. A loop with both pyrimidine and purine nucleotides is described as a mix. The IRES database and thermodynamic database contain 107 and 469 total loops, respectively. Note that the thermodynamic database does not include the new measurements presented in this work. For comparisons of loop size, 1 × 2 loops are shown in blue; 1 × 3 loops in peach; 1 × *n* loops in green; 2 × 2 loops in black; 2 × 3 loops in gray; 2 × *n* loops in bright pink; 3 × 3 loops in yellow; 3 × *n* loops in purple; and *n* × *n* loops in pastel pink. For comparisons of loop symmetry, symmetric loops are shown in blue, and asymmetric loops are shown in pink. For comparisons of nucleotide content, purine-only loops are shown in black, pyrimidine-only loops in gray; and loops with a mix of purine and pyrimidines in green.

All the thermodynamic data incorporated in the 2004 prediction model (Mathews et al. 2004) were compiled and updated with new data for 126 RNA loops (Chen et al. 2004, 2006, 2009; Bourdelat-Parks and Wartell 2005; Badhwar et al. 2007; Christiansen and Znosko 2008, 2009; Hausmann and Znosko 2012; Zhong et al. 2015). A complete spreadsheet of loop thermodynamic parameters is available in the Supplemental Information. Prior to this study, the thermodynamic database contained a total of 469 internal loops. The IRES database consists of 107 internal loops from viruses and eukaryotic mRNAs whose RNA structures have been determined through chemical modification and phylogenetic analysis (Mokrejs et al. 2010). Analysis of the databases compared the size of the loops, the nucleotide content within the loops, and loop symmetry (Fig. 1). Forty-two percent of the thermodynamic database consisted of 2 × 2 loops. In contrast, 2 × 2 loops only comprised 15% of the IRES database. The IRES database had a more even distribution in terms of loop size. Loops with >6 nt were the largest category in the IRES database (17% *n* × *n* loops where *n* is greater than three). Fifty percent of the loops within the thermodynamic database contained only purines. In contrast, only 14% of the loops within the IRES database were purines. Sixty-two percent of the loops were a mixture of purines and pyrimidines in the IRES database. Lastly, the distribution of symmetric and asymmetric loops in both databases was completely opposite. Sixty-five percent of the loops within the thermodynamic database were symmetric, primarily due to the substantial number of 2 × 2 loops. In contrast, 72% of the IRES loops were asymmetric. The differences between the two databases may contribute to the limited accuracy of predictions for viral RNA structures (Mathews et al. 2004). Additional thermodynamic data for large, asymmetric pyrimidine-rich loops will improve predictions for viral IRES RNA.

### New thermodynamic data diversifies the database

In order to expand the thermodynamic database to better represent loops that occur in virus IRES, additional thermodynamic data on RNA duplexes containing large, asymmetric, pyrimidine loops were collected. Table 1 shows the optical melting data of internal loops that are pyrimidine rich and that have sequences modeled on viral IRES structures. Both the linear van't Hoff plot and melt curve fits data in Table 1 are shown in order to validate two-state behavior. Thermodynamic data for internal loops studied at pH 5.5, a pH at which cytidines become protonated, are listed below the data for pH 7 buffer conditions.

**TABLE 1.**
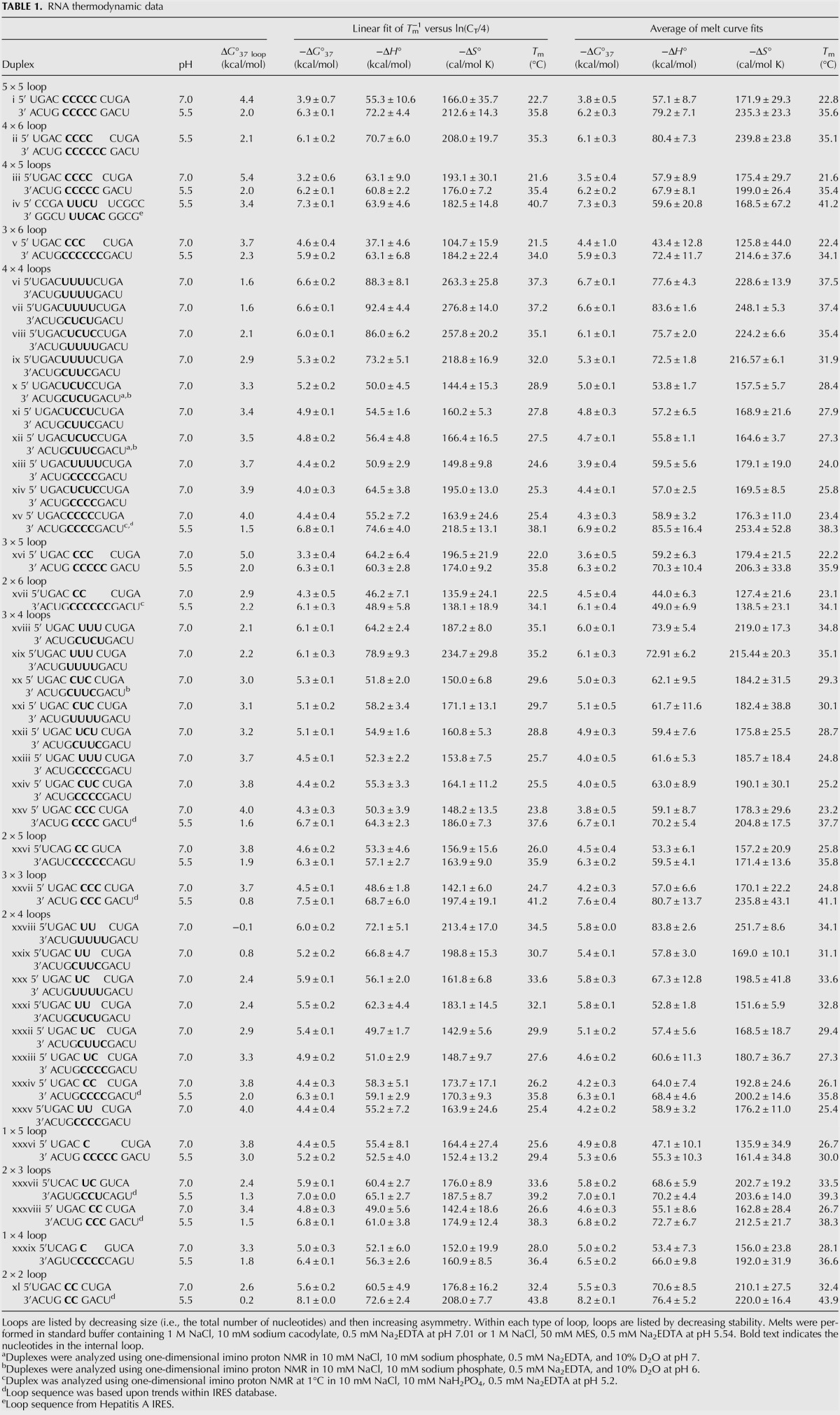
RNA thermodynamic data

As shown in Table 1 column 3, loops with the potential to form UU pairs are more stable than those without possible UU pairs at pH 7. Furthermore, loops that contain more than one possible UU pair are more stable than loops that only contain one possible pair. The range of free energies for all the new loops studied was −0.1 kcal/mol to 5.4 kcal/mol. In general, loop stability decreased with increasing loop size, and uracil loops were discovered to be more stable than cytidine loops of the same size. For example, the measured free energy at 37°C of the 3 × 4 all-uracil loop was found to be 2.16 kcal/mol in contrast to the 3 × 4 all-cytidine loops that had a free energy value of 4.0 kcal/mol. The 2 × 4 uracil loop (duplex xviii) was the most stable of all the loops studied, with a free energy of −0.1 kcal/mol. Thus, even though loop stability generally becomes less favorable with increasing loop size and asymmetry, UU pairs provide adequate stability to overcome the energetic costs of larger, asymmetric loops. For example, the largest all-uracil 4 × 4 loop (Δ*G*_loop_ = 1.6 kcal/mol, duplex vi) is more stable than the smallest all-cytidine 2 × 2 loop (Δ*G*_loop_ = 2.6 kcal/mol, duplex xl).

The loop free energies determined experimentally were then compared to current predicted values (Supplemental Table 1; Mathews et al. 2004). The current prediction model generally overpredicts the stabilities of pyrimidine loops at pH 7. The predicted loop free energies for 13 of the 40 new loops measured at pH 7 are different from experimental values by >1.0 kcal/mol, which is greater than the experimental error range of ±0.5 kcal/mol. The largest prediction error is 2.5 kcal/mol for an all cytidine 4 × 5 loop (duplex iii), which is a larger loop than any previous loops measured. Thus, these new measurements of internal loops will improve the accuracy of future predictions and diversify the thermodynamic database.

### Uracil base-pairing contributes to loop stability

Although the 2004 prediction model (Mathews et al. 2004) gives a bonus for UU pairing that occurs adjacent to closing base pairs, it does not account for UU mismatches that can occur across or within the middle of asymmetric internal loops. In order to determine whether UU base-pairing occurs within internal loops, sequences containing potential UU mismatches within internal loops were designed and studied through optical melting and one-dimensional imino proton NMR. For example, some of the loops tested if UU noncanonical pairing would occur across asymmetric internal loops. The duplex 5′UGAC UUU CUGA/3′ACUG UUUU GACU (duplex xix) has the potential for a total of three noncanonical UU pairs and a free energy of 2.2 kcal/mol, whereas 5′UGAC CUC CUGA/3′ACUG CUUC GACU (duplex xx) has the potential for one UU pair and has a free energy of 3.0 kcal/mol.

### NMR indicates UU noncanonical pairs occur across asymmetric internal loops

One-dimensional imino proton experiments were then performed to test if UU nucleotides form hydrogen-bonded pairs across asymmetric internal loops. All of the oligonucleotides studied by NMR have identical stems, and thus four GC and two AU imino protons are expected in all of the NMR spectra. UU imino protons resonate around 10.4–11.3 ppm (Schroeder and Turner 2000) and thus peaks that occur within this region can be attributed to UU base-pairing. In Supplemental Figure 1, four of the five duplexes that have the potential to form UU pairs show peaks in this region. The loop sequence 5′CUUC/3′GCCCCG (duplex xxxv) has no potential UU pairs and thus serves as the control and helps assign imino proton resonances in the stem Watson–Crick pairs. At least seven of the eight expected UU imino peaks were observed in the spectra of the 4 × 4 all-uridine loop (duplex xxviii) (Supplemental Fig. S1), which suggests multiple UU hydrogen-bonded pairs form in this loop. This 4 × 4 all-uridine loop is also among the most thermodynamically stable loops. The imino proton spectra demonstrate the formation of stable hydrogen-bonded UU pairs that can occur adjacent to and also nonadjacent to the closing Watson–Crick pair of the loop. These NMR data support the free energy bonus term for loops with the potential to form UU pairs (see Discussion below).

### Protonated cytosine pairs stabilize internal loops

**FIGURE 2.**
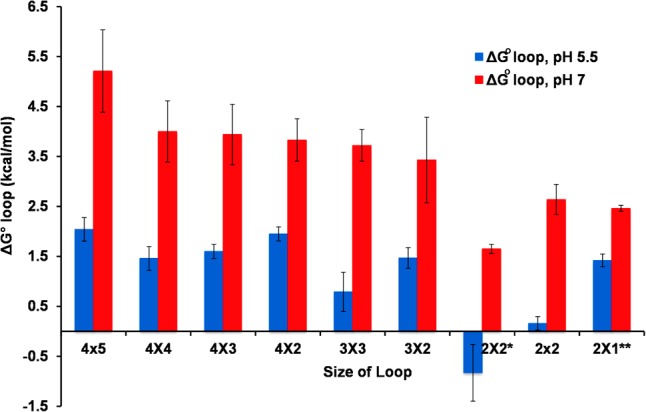
Loops with all cytidine nucleotides are more stable at pH 5. Comparison of cytidine loop free energies at pH 7 (red bars) and pH 5 (blue bars). Standard error was determined by comparing the free energy errors from the linear and melt curve fits. (*) Data obtained from Santa Lucia et al. (1991). The duplex sequence is 5′CGC CC GCG/3′GCG CC CGC and has only 3 bp in the stems. The difference in the length of the surrounding stems may explain the difference in stabilities for this loop (Schroeder and Turner 2000). (**) Data obtained from Schroeder and Turner (2000).

As shown in Figure 2, an acidic environment stabilizes internal cytidine loops. The range of free energies for all cytidine loops is 5.2 to 2.6 kcal/mol and 2.3 to 0.2 kcal/mol at pH 7 and 5.5, respectively. The stability of the cytidine loops at low pH may be due to the greater potential for protonated cytosine pairs to form an additional hydrogen bond and/or changes in base stacking. For protonated cytosine pairs, the expected resonance frequencies occur between 10.4 and 11.3 ppm (Santa Lucia et al. 1991). Other than 2 × 2 loops, no additional imino protons were observed within the regions where protonated cytidines resonate for loops. Thus, the added stability of cytosine base pairs at low pH may be due more to base stacking interactions than hydrogen bonding.

Symmetric and asymmetric loops show different stabilizing effects at pH 5.5. In general, symmetric loops are more stable than asymmetric loops, and smaller loops are more stable than larger loops at pH 7, which is consistent with previous studies of adenine loops (Peritz et al. 1991) and cytidine loops (Weeks and Crothers 1993). The asymmetric cytidine loops, however, show much less dependence on size and asymmetry at pH 5.5. The range of loop free energies for asymmetric cytidine loops is only 2.2 to 1.5 kcal/mol. A difference of only 0.7 kcal/mol is near the experimental error in optical melting measurements and calculation of loop free energies. Thus, the weighted average free energy of the asymmetric cytidine loops at pH 5.5 is 2.0 kcal/mol and provides a reasonable prediction value. The proposed prediction rule for symmetric cytidine loops at pH 5.5 is to subtract 2.5 kcal/mol from the loop free energy at pH 7, which maintains the dependence on loop size. The symmetric loops also show a >10 kcal/mol change in loop enthalpy at lower pH, which would be consistent with changes in base stacking and hydrogen bonding.

Interestingly, a 4 × 5 asymmetric loop, 5′AUUCUU3′/3′UUUCACG5′ (duplex iv), based on a loop sequence in the Hepatitis A IRES (Mokrejs et al. 2010), shows a clear two-state transition only at pH 5.5 and not at pH 7. Similarly, a 4 × 6 all-cytidine loop (duplex ii) shows two-state melting behavior only in pH 5.5 conditions. The IRES loop has possible protonated C^+^C^+^ pairs and C^+^U pairs as well as UU pairs. This 4 × 5 hepatitis IRES loop (3.4 kcal/mol, duplex iv) is less stable than the 4 × 5 all-cytidine loop (2.0 kcal/mol, duplex iii), however. Thus, the additional stability of the possible U–U pairs may be less than the protonated cytosine pairs at pH 5.5. The different stabilities for IRES loops at different pH conditions may facilitate conformational changes in viral RNA that are triggered by pH changes, such as virus disassembly.

### Proposed model for predicting the free energies of asymmetric internal loops

Optical melting data were analyzed with the linest function in Excel to obtain new prediction rules for asymmetric loops. Factors considered in the prediction rules include loop size, symmetry, or asymmetry, AU or GU closing base pairs, and potential GA, GG, UU, or protonated C^+^C^+^ pairs (Gralla and Crothers 1973). Other models for loop stability with different parameters were analyzed but did not produce better results. The current prediction model (2004 model, Mathews et al. 2004) is depicted in Equation 1 below:

(1) $$\Delta G_{\mathrm{loop}}^{\circ }=\Delta G_{\text{loop initiation}}^{\circ }+\Delta G_{\text{asymmetry penalty}}^{\circ }+\Delta G_{\text{AU/GU penalty}}^{\circ }+\Delta G_{\text{GG/GA/UU bonus}}^{\circ }.$$

The last free energy term is used to account for GG, GA, or UU pairs that occur adjacent to the closing Watson–Crick pairs. Analysis of the new data suggests the current model should be revised to include UU, GG, and GA base pairs that occur across internal loops and an additional term for protonated cytosine base pairs. For loops that were capable of either forming a GA or GG pair, preference was given to GA pairs due to their increased thermodynamic stability in comparison to GG pairs on average over all loop types. A revised model (2016) is proposed in Equation 2 and Table 2:

(2) $$\Delta G_{\mathrm{loop}}^{\circ }=(\Delta G_{\text{loop initiation}}^{\circ }+\Delta G_{\text{asymmetry penalty}}^{\circ })+\Delta G_{\text{AU/GU penalty}}^{\circ }+\Delta G_{\mathrm{GG}/\mathrm{GA}/\mathrm{UU}/C^{+}C^{+}\;\mathrm{bonus}}^{\circ }.$$

**TABLE 2.**
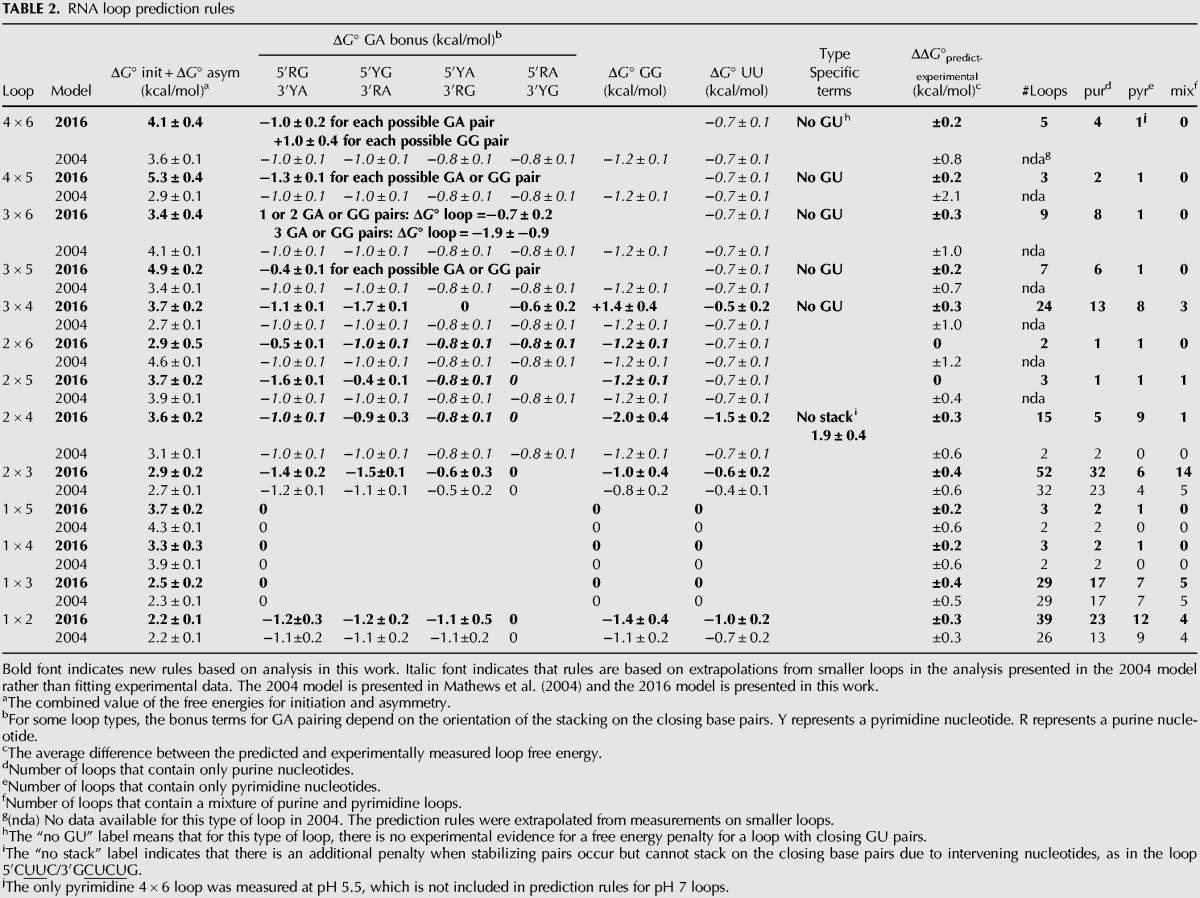
RNA loop prediction rules

Table 2 lists one parameter for loop initiation and asymmetry because each type of loop was analyzed separately using linear regression. Thus, both the loop initiation and asymmetry are a single constant value in the linear regression analysis. For example, in order to predict the stability of the 3 × 4 loop 5′CCUCC/3′GCUUCG at pH 7 (duplex xx), add together 3.7 kcal/mol (Δ*G*_loop_ + Δ*G*_asymmetry_), 0.0 kcal/mol (Δ*G*_AU/GU penalty_), and −0.5 kcal/mol (Δ*G*_UU bonus_). Using the new parameters for stabilizing pairs across an internal loop and revised initiation terms for larger sized loops, the prediction of the experimental free energies for asymmetric loops is within 0.3 kcal/mol on average. Sequence-dependent prediction rules for symmetric loops are still under development in collaboration with the Turner laboratory and alumni. Table 2 summarizes the improvements in the prediction parameters for asymmetric loops, database size, and database diversity between the 2004 model and the 2016 model.

**FIGURE 3.**
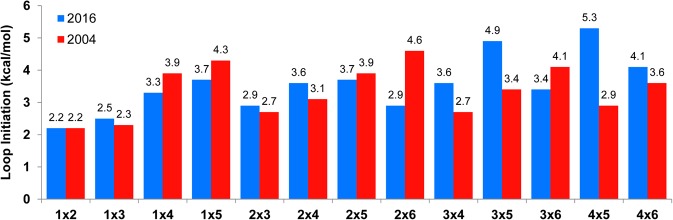
Loop initiation terms in the 2016 and 2004 models. Loop initiation parameters include the terms for the free energy penalties for loop asymmetry. The parameters are the constant value in the linear regression analysis of each type of loop.

New initiation terms for asymmetric loops are proposed in Table 2. Linear regression analysis of purine and pyrimidine loops separately revealed there are no biases in the loop initiation and asymmetry terms. The 2004 model used an asymmetry penalty of 0.7 kcal/mol per degree asymmetry and the Jacobson–Stockmayer approximation to estimate the free energies of initiation for larger, unmeasured loops. This approximation does not predict well the new loop measurements presented in Table 1 and Supplemental Table 1. Figure 3 shows that this approximation both overpredicts and underpredicts the cost of loop initiation. For example, in loops with 8 nt, the 2016 and 2004 parameters are 2.9 and 4.6 kcal/mol for 2 × 6 loops and 4.9 and 3.4 kcal/mol for 3 × 5 loops, respectively. The new initiation terms for 2 × 6 and 3 × 5 loops, which have asymmetries of four and two, respectively, do not follow the previous assumption that increasing asymmetry destabilizes a loop. Similarly for loops of 9 nt, the 3 × 6 and 4 × 5 loop initiation terms are 3.4 and 5.3 kcal/mol, respectively, for loops with three and one asymmetries, respectively. Thus, the occurrence of large, highly asymmetric internal loops in virus IRES may not be as energetically unfavorable as previously predicted.

The 2016 model retains the value of −0.7 kcal/mol for an AU closing base pair. This parameter repeatedly gave the same value within error for all linear regression analyses. The value of −0.7 kcal/mol is consistent with measurements of the free energy of a hydrogen bond and accounts for the difference in the number of hydrogen bonds in AU and GC pairs (Xia et al. 1998). The same value has been used for loops with closing GU pairs (Schroeder and Turner 2001). In this analysis of 4 × 6, 4 × 5, 3 × 6, 3 × 5, and 3 × 4 loops, however, there was little evidence for any penalty for loops with GU pairs. For example, the free energies of 3 × 5 loops 5′UGAAG/3′GAAGGAC and 5′CGAAG/3′GAAGGAC are both 1.8 kcal/mol, and the free energies of 3 × 4 loops 5′CGAAG/3′GAAGGC and 5′UGAAG/3′GAAGGC are −0.7 and −0.5 kcal/mol, respectively (Chen et al. 2006). The thermodynamic stabilities of GU pairs are highly idiosyncratic (Schroeder and Turner 2001; Schroeder et al. 2003; Nguyen and Schroeder 2010; Chen et al. 2012), and in some contexts, the stacking interactions with GU pairs may outweigh the loss of a hydrogen bond.

Table 2 shows how the values for the bonus terms for GA, GG, and UU pairs vary with different loop sizes and asymmetries. The bonus values for GA pairs depend on the orientation and stacking of the GA pair. For example, in 2 × 3 loops, a 5′RG/3′YA pair adds −1.4 kcal/mol while a 5′RA/3′YG pair adds no stability. (Note that R and Y indicate a purine or a pyrimidine in a Watson–Crick pair, respectively.) A 5′RA/3′YG in a 2 × 4 loop and a 5′YA/3′RG pair in a 3 × 4 loop also add no stability to a loop. The range of additional stabilities provided by GA pairs in an asymmetric loop ranges from zero to −1.8 kcal/mol depending on the loop size, asymmetry, and base stacking. For 5 × 5, 4 × 6, 4 × 5, 3 × 6, and 3 × 5 loops, the 2004 model clearly does not predict well the experimental values, but there are less than 10 loop measurements for each type of loop. In these cases, there are too few measurements to parse statistically significant differences between different GA stacking interactions, and an average value is used for the prediction rule.

The GG bonus varies widely between different loops. In 2 × 4 loops, a GG pair adds −2.0 kcal/mol while a GG pair in a 3 × 4 loop is destabilizing by 1.4 kcal/mol. Guanine nucleotides have many hydrogen bond donors and acceptor sites and are able to form many different types of pairs (Leontis et al. 2002). Guanine nucleotides also have the largest dipole moment (Bloomfield et al. 2000) and thus are capable of forming strong stacking interactions. Thus, the thermodynamic stabilities of GG pairs are highly idiosyncratic (Burkard and Turner 2000).

Uridine nucleotides have weaker stacking interactions than guanine nucleotides and show less variation in the bonus for UU pairs (Freier et al. 1983). The values for UU bonus terms range from −0.4 kcal/mol to −1.0 kcal/mol for all loops except 2 × 4 loops. The 2 × 4 loop prediction rule also includes a penalty for when the formation of possible noncanonical stabilizing pairs occurs in ways that would force nucleotides to bulge out of the loop if the noncanonical pair stacked on the closing Watson–Crick pair. Individual bulge nucleotides also have an unfavorable parameter in prediction rules, and similar unfavorable energetic effects of backbone distortion may occur in small highly asymmetric loops such as 2 × 4 loops.

The 2016 model now includes a bonus term for protonated CC pairs in symmetric loops at pH 5 that would account for stabilizing hydrogen bonding and stacking interactions. Some viruses, such as enteroviruses, can survive in acidic environments and the release of viral genomes into the host cell is mediated by a change in pH. For these reasons, the cytidine loops were studied at both pH 7.0 and 5.5. The new prediction rule for loops with protonated cytosine pairs depends on whether the loop is symmetric or asymmetric. The symmetric cytidine loops show more dependence on loop size. Recent crystal structures of tandem CC pairs (2 × 2 loop) at neutral pH show stacking interactions and a single hydrogen bond between the N3 and amino groups or a bifurcated hydrogen bond from the amino group to the N3 and the carbonyl (Dodd et al. 2016). This type of pairing would not be possible if the N3 position were protonated, but possible C^+^C^+^ pairs could form with two hydrogen bonds. There are several possible conformations for CC and CU pairs that would be disrupted by cytosine protonation (Kiliszek et al. 2012; Rypniewski et al. 2016). Cytidine protonation would also change stacking interactions. A cytidine tetramer shows A-form stacking interactions by NMR (Tubbs et al. 2013). If the cytidines were protonated, then different stacking interactions would likely form. The stabilities of stacking interactions for cytidines in the unfolded single strand state are likely similar to stacking interactions for adenines because the similarities of loop stabilities for all-adenine loops and all-cytidine loops at neutral pH do not have any stabilizing bonus terms.

### Conclusion

The thermodynamic database for asymmetric internal loops has been curated and expanded. The new initiation terms for loops containing >6 nt greatly improved accuracy in predicting the experimental free energies. The stabilities of large, highly asymmetric internal loops that occur in virus IRES may be more favorable than previously predicted. The bonus terms for GA, GG, UU, and protonated CC pairs vary with the size and asymmetry of the loop. The thermodynamics obtained from optical melting experiments suggests that protonated cytosines and uracil base-pairing at any position in internal loops contribute to loop stability. One-dimensional imino proton NMR spectra validate the formation of UU base pairs, even when the pairs may not stack directly on the closing Watson–Crick pairs. The bonus terms for cytidine loops at low pH will improve predictions for viral RNA that undergo conformational changes in different pH environments. These results will contribute to the ongoing effort to expand and diversify the RNA thermodynamic database and improve RNA structure prediction.

## MATERIALS AND METHODS

The IRES structures studied were obtained from iresite.org (Mokrejs et al. 2010), which contains a database of experimentally determined IRES structures. The database contains IRES structures from 11 viruses and 11 eukaryotic cellular mRNAs that contain a total of 107 IRES internal loops. Although IRESs are also rich in bulge loops, hairpins, and pseudoknots, only internal loops were examined for this study.

RNA oligos were obtained from Dharmacon and then deblocked according to the manufacturer's protocol. The concentrations of the oligos were then determined by measuring the absorbance at 80°C and 260 nm using a Beckman DU800 spectrophotometer with temperature control. The RNA oligos were then dissolved in standard optical melting buffer, which consists of 1 M NaCl, 0.5 mM Na_2_EDTA, and 10 mM sodium cacodylate or 50 mM MES at pH 7.01 or 5.54, respectively. One-dimensional imino proton NMR data were collected with samples at 0.5 mM RNA concentrations in NMR buffer containing 10 mM NaCl, 10 mM potassium phosphate in 90% H_2_O, and 10% D_2_O at pH's 6.0 and 4.5 with Watergate solvent suppression on a 500 MHz Varian NMR spectrometer (Piotto et al. 1992; Lukavsky and Puglisi 2001).

Single-stranded melts were performed to check if stable homodimers form. For both single strand and heteroduplex melts, the absorbance at 260 nm was measured as a function of temperature from 0.1°C to 80°C at a heating rate of 1°C/min. The individual melt curves were fit using Meltwin software (McDowell and Turner 1996). A plot of the Tm^−1^ versus the natural log of the duplex concentration was fit according to the equation:

(3) $$1/T_{m}=-(R/\Delta H^{\circ })\ln\left(\mathrm{conc}.\right)+\Delta S^{\circ }/\Delta H^{\circ },$$

where R is the gas constant (Schroeder and Turner 2009). If the enthalpy terms agree within 15% between the two different fits, then the heteroduplex is considered to be two-state. Using the equation from the Van't Hoff plot in conjunction with the Gibbs free energy Equation 1, the thermodynamic parameters ΔG, ΔH, and ΔS can be determined for the duplex. Using the nearest-neighbor approximations (Xia et al. 1998), the free energy of the stems can be calculated and subtracted from the duplex to get the free energy of the loop itself.

(4) $$\Delta G_{\mathrm{loop}}^{\circ }=\Delta G_{\mathrm{duplex}}^{\circ }-\Delta G_{\mathrm{stems}}^{\circ }+\Delta G_{\mathrm{interrupted}\;\mathrm{nearest}\;\mathrm{neighbor}}^{\circ }.$$

For example, in order to calculate the experimental free energy for the 3 × 4 loop 5′CCUCC/3′GCUUCG at pH 7, add −5.3 kcal/mol (Δ*G*_duplex_ 5′UGAC CUC CUGA/3′ACUG CUUC GACU), −11.5 (Δ*G*_stems_ 5′UGACCUGA/3′ACUGGACU), and −3.3 kcal/mol (Δ*G*_interrupted nearest neighbor_ 5′CC/3′GG).

Data from optical melting experiments were fit using the linest function in Excel. The loops at pH 7 from this work were combined with all previous measurements of internal loop stabilities (Santa Lucia et al. 1990, 1991; Peritz et al. 1991; Walter et al. 1994; Wu et al. 1995; Schroeder et al. 1996; Xia et al. 1997; Schroeder and Turner 2000, 2001; Burkard et al. 2001; Chen et al. 2004, 2005, 2006, 2009; Bourdelat-Parks and Wartell 2005; Chen and Turner 2006; Badhwar et al. 2007; Christiansen and Znosko 2008, 2009; Hausmann and Znosko 2012; Zhong et al. 2015). Thermodynamic data were manually curated, and data with non-two-state behavior, highly probable alternate duplex structures, and three or more consecutive guanine nucleotides without single-strand optical melting data were not included in the database for analysis. The prediction model was assessed by the number of loops that were predicted within 0.3 kcal/mol and 0.5 kcal/mol; low standard deviation in the model parameters; and Students’ *t* and *P* tests. Loops were grouped by size and asymmetry, and new prediction rules were tested for each loop type.

## SUPPLEMENTAL MATERIAL

Supplemental material is available for this article.

## Supplementary Material

Supplemental Material
