# Supplementary material for: Advancing viral RNA structure prediction: measuring the thermodynamics of pyrimidine-rich internal loops
Source: RNA. 2017 May;23(5):770–81. doi: 10.1261/rna.059865.116 (PMC5393185; doi:10.1261/rna.059865.116)
Supplement: Supplemental Material [file supp_23_5_770__index.html]

Advancing viral RNA structure prediction: measuring the thermodynamics of pyrimidine-rich internal loops — Supplemental Material 

# Advancing viral RNA structure prediction: measuring the thermodynamics of pyrimidine-rich internal loops

## Supplemental Material

- Supplemental\_Figure\_Table.docx
- Supplemental\_thermodatabase.xlsx
