# Supplementary material for: Advancing viral RNA structure prediction: measuring the thermodynamics of pyrimidine-rich internal loops
Source: RNA. 2017 May;23(5):770–81. doi: 10.1261/rna.059865.116 (PMC5393185; doi:10.1261/rna.059865.116)
Supplement: Supplemental Material [file supp_059865.116_Supplemental_Figure_Table.docx]

**Supporting Information for**

**Advancing viral RNA structure prediction: measuring the thermodynamics of pyrimidine-rich internal loops**

Andy Phan^†^, Katherine Mailey^†^, Jessica Sakai^†^, Xiaobo Gu, Susan J. Schroeder*

^†^ These authors contributed equally to this work.

*Corresponding author

Supporting Information includes a table of experimental and predicted loop free energies, one-dimensional imino proton NMR, and an Excel file of 194 loop free energy values.

**Supporting Information Table S1**.

Comparison of Experimental and Predicted Loop Free Energy

| Sequence | ΔG˚_37 Loop Exp_  (kcal/mol) | ΔG˚_37 loop predict_  (kcal/mol) | ∆ΔG˚_37_  (kcal/mol) |
| --- | --- | --- | --- |
| **5x5 Loop** |  |  |  |
| 5’ UGAC **CCCCC** CUGA  3’ACUG **CCCCC** GACU | 4.4 | 2.5 | 1.9 |
| **4x5 Loop** |  |  |  |
| 5’UGAC **CCCC** CUGA  3’ACUG **CCCCC** GACU | 5.4 | 2.9 | 2.5 |
| **3x6 Loop** |  |  |  |
| 5’ UGAC **CCC** CUGA  3’ ACUG **CCCCCC** GACU | 3.7 | 4.1 | -0.4 |
| **4x4 Loops** |  |  |  |
| 5’ UGAC**UUUU**CUGA  3’ ACUG**UUUU**GACU | 1.6 | 0.8 | 0.8 |
| 5’ UGAC**UUUU**CUGA  3’ ACUG**CUCU**GACU | 1.6 | 1.5 | 0.1 |
| 5’ UGAC**UCUC**CUGA  3’ ACUG**UUUU**GACU | 2.1 | 1.5 | 0.6 |
| 5’ UGAC**UUUU**CUGA  3’ ACUG**CUUC**GACU | 2.9 | 2.2 | 0.7 |
| 5’ UGAC**UCUC**CUGA  3’ ACUG**CUCU**GACU | 3.3 | 2.2 | 1.1 |
| 5’ UGAC**UCCU**CUGA  3’ ACUG**CUUC**GACU | 3.4 | 2.2 | 1.2 |
| 5’ UGAC**UCUC**CUGA  3’ ACUG**CUUC**GACU | 3.5 | 2.2 | 1.3 |
| 5’ UGAC**UUUU**CUGA  3’ ACUG**CCCC**GACU | 3.7 | 2.2 | 1.5 |
| 5’ UGAC**UCUC**CUGA  3’ ACUG**CCCC**GACU | 3.9 | 2.2 | 1.7 |
| 5’ UGAC**CCCC**CUGA  3’ ACUG**CCCC**GACU | 4.0 | 2.2 | 1.8 |
| **3x5 Loop** |  |  |  |
| 5’ UGAC **CCC** CUGA  3’ ACUG **CCCCC** GACU | 5.0 | 3.4 | 1.6 |
| **2x6 Loop** |  |  |  |
| 5’ UGAC **CC** CUGA  3’ ACUG **CCCCCC** GUCA | 2.9 | 4.6 | -1.7 |
| **3x4 Loops** |  |  |  |
| 5’ UGAG **UUU** CUGA  3’ ACUG**CUCU**GACU | 2.1 | 2.0 | 0.1 |
| 5’ UGAC **UUU** CUGA  3’ ACUG**UUUU**GACU | 2.2 | 1.3 | 0.9 |
| 5’ UGAC **CUC** CUGA  3’ ACUG**CUUC**GACU | 3.0 | 2.7 | 0.3 |
| 5’ UGAC **CUC** CUGA  3’ ACUG**UUUU**GACU | 3.1 | 2.7 | 0.4 |
| 5’ UGAC **UCU** CUGA  3’ ACUG**CUUC**GACU | 3.2 | 2.7 | 0.5 |
| 5’ UGAC **UUU** CUGA  3’ ACUG**CCCC**GACU | 3.7 | 2.7 | 1.0 |

| Sequence ^a^ | ΔG˚_37 Loop Exp_  (kcal/mol)^b^ | ΔG˚_37 predict_  (kcal/mol)^c^ | ∆ΔG˚_37_  (kcal/mol)^f^ |
| --- | --- | --- | --- |
| **3x4 Loops** continued |  |  |  |
| 5’ UGAC **CUC** CUGA  3’ ACUG**CCCC**GACU | 3.8 | 2.7 | 1.1 |
| 5’ UGAC **CCC** CUGA  3’ ACUG**CCCC**GACU | 3.9 | 2.7 | 1.2 |
| **2x5 Loop** |  |  |  |
| 5’UCAG  **CC** GUCA  3’AGUC**CCCCC**CAGU | 3.8 | 3.9 | -0.1 |
| **2x4 Loops** |  |  |  |
| 5’ UGAC **UC** CUGA  3’ ACUG**UUUU**GACU | 2.3 | 2.4 | -0.1 |
| 5’ UGAC **UU** CUGA  3’ ACUG**CUCU**GACU | 2.5 | 2.4 | 0.1 |
| 5’ UGAC **UU** CUGA  3’ ACUG**UUUU**GACU | 2.5 | 2.4 | 0.1 |
| 5’ UGAC **UU** CUGA  3’ ACUG**CUUC**GACU | 2.8 | 3.1 | -0.3 |
| 5’ UGAC **UC** CUGA  3’ ACUG**CUUC**GACU | 2.9 | 3.1 | -0.2 |
| 5’ UGAC **UC** CUGA  3’ ACUG**CCCC**GACU | 3.3 | 3.1 | 0.2 |
| 5’ UGAC **CC** CUGA  3’ ACUG**CCCC**GACU | 3.8 | 3.1 | 0.7 |
| 5’ UGAC **UU** CUGA  3’ ACUG**CCCC**GACU | 4.0 | 3.1 | 0.9 |
| **1x5 Loop** |  |  |  |
| 5’ UGAC **C** CUGA  3’ ACUG **CCCCC** GACU | 3.8 | 4.3 | -0.5 |
| **2x3 Loops** |  |  |  |
| 5’UCAC **UC** GUCA  3’AGUG**CCU**CAGU | 2.4 | 2.7 | -0.3 |
| 5’ UGAC **CC** CUGA  3’ACUG **CCC** GACU | 3.4 | 2.7 | 0.7 |
| **1x4 Loop** |  |  |  |
| 5’UCAG **C** GUCA  3’AGUC**CCCC**CAGU | 3.3 | 3.9 | -0.6 |
| **2x2 Loop** |  |  |  |
| 5’UGAC **CC** CUGA  3’ACUG **CC** GACU | 2.6 | 1.1 | 1.5 |

**Supporting InformationTable S1**: Sequences are ordered by size of internal loop (4x4, 3x4, 2x4) then by decreasing thermodynamic stability. Bold and underlined letters represent the internal loop. RNA duplexes were melted in 1 M NaCl, 10 mM sodium cacodylate, 0.5 mM Na_2_EDTA buffer at pH 7.01. Experimental free energy values are calculated with values from the plots of T_M_^-1^ vs. ln(C_T_/4) and equation 4. Predicted free energy values are calculated using the model from ([Mathews 2004](#_ENREF_25)) . ∆∆G° calculated from ∆G°_loop exp_ - ∆G°_loop pred_. Positive value means stability was over predicted.

**Supporting Information Figure S1**


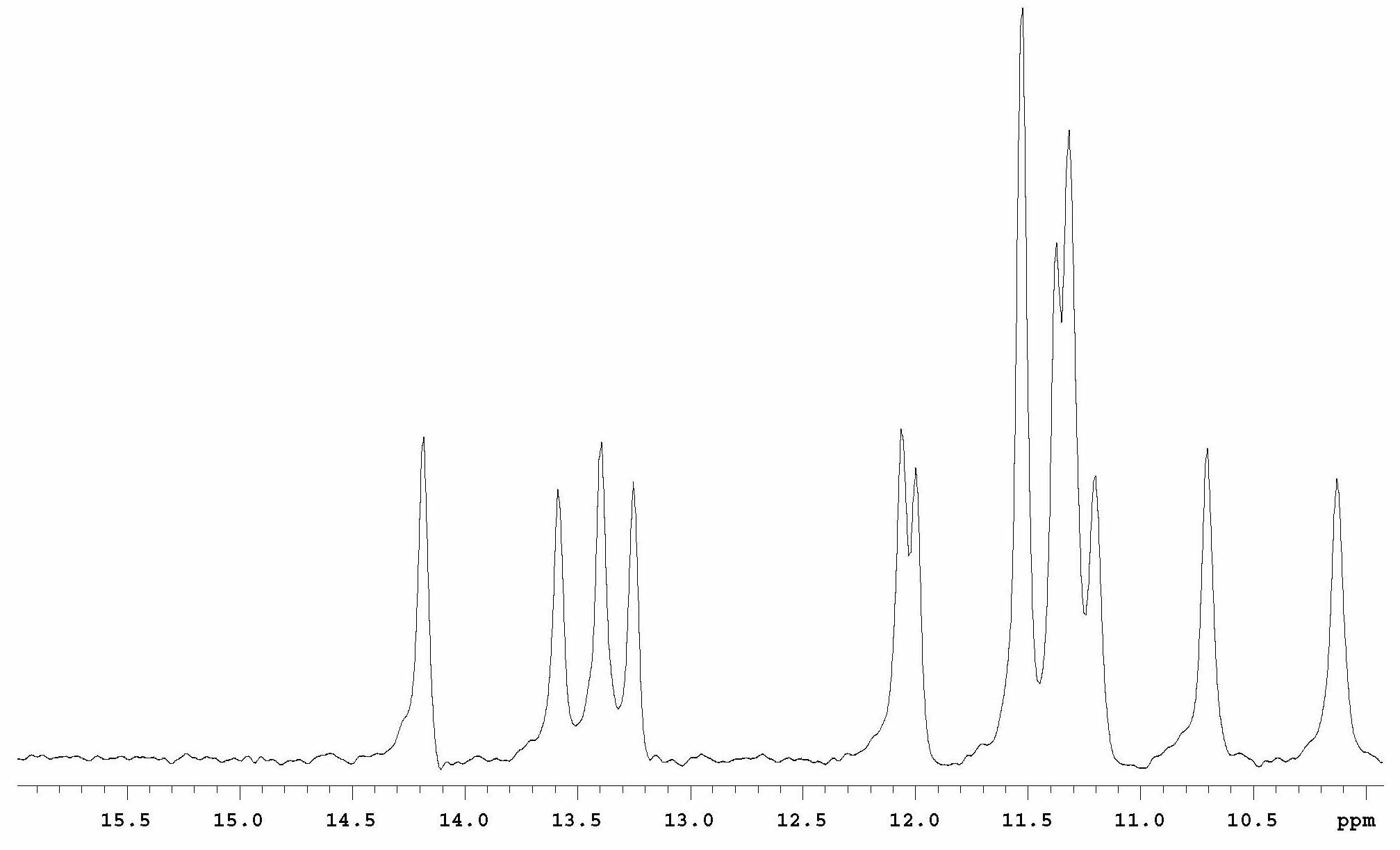

$$\begin{matrix} 5^{'}\mathrm{UGAC}\mathbf{UUUU}\mathrm{CUGA} \\ 3^{'}\mathrm{ACUG}\mathbf{UUUU}\mathrm{GACU} \end{matrix}$$

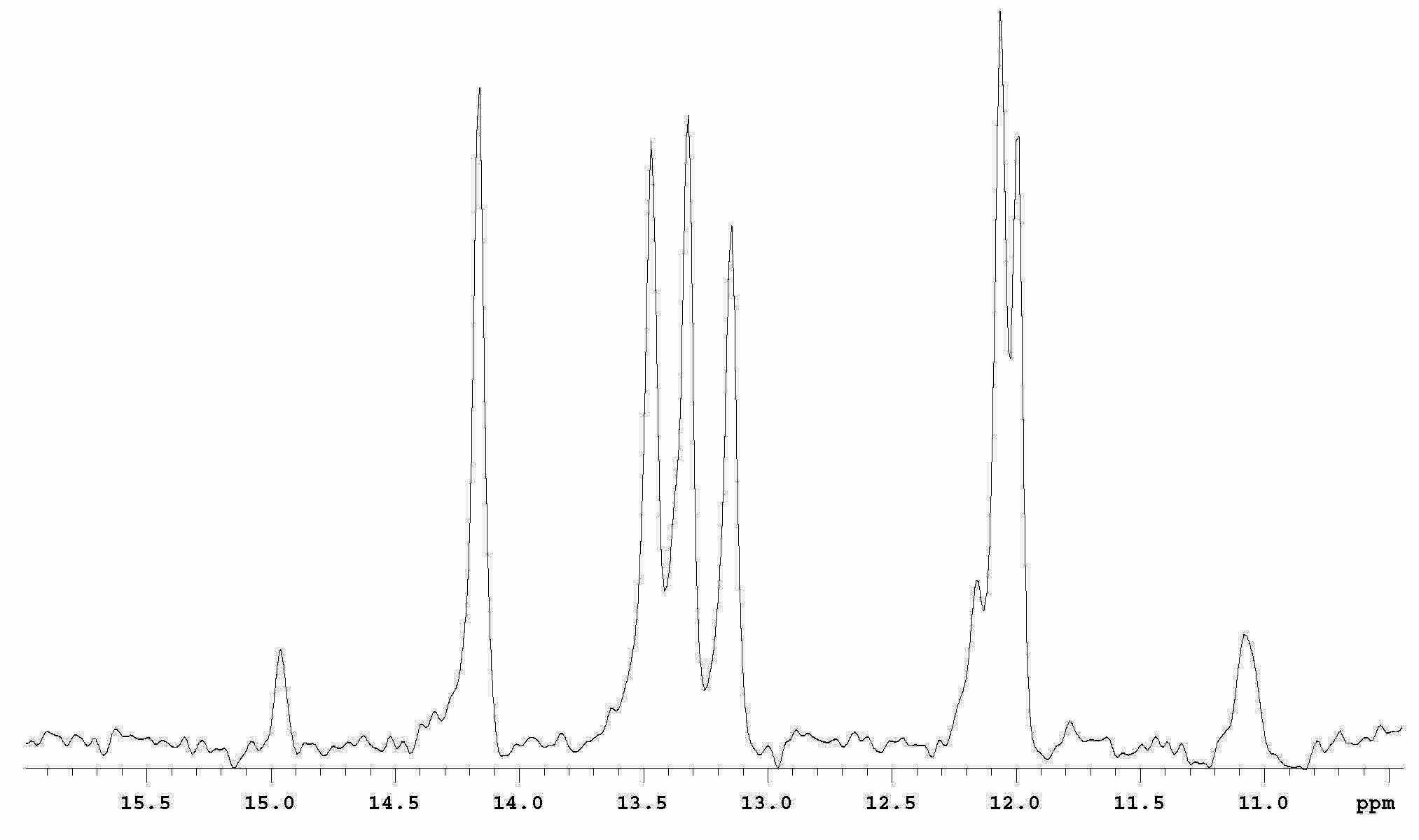

$$\begin{matrix} 5^{'}\mathrm{UGAC}\mathbf{CUC}\mathrm{CUGA} \\ 3^{'}\mathrm{ACUG}\mathbf{CUUC}\mathrm{GACU} \end{matrix}$$

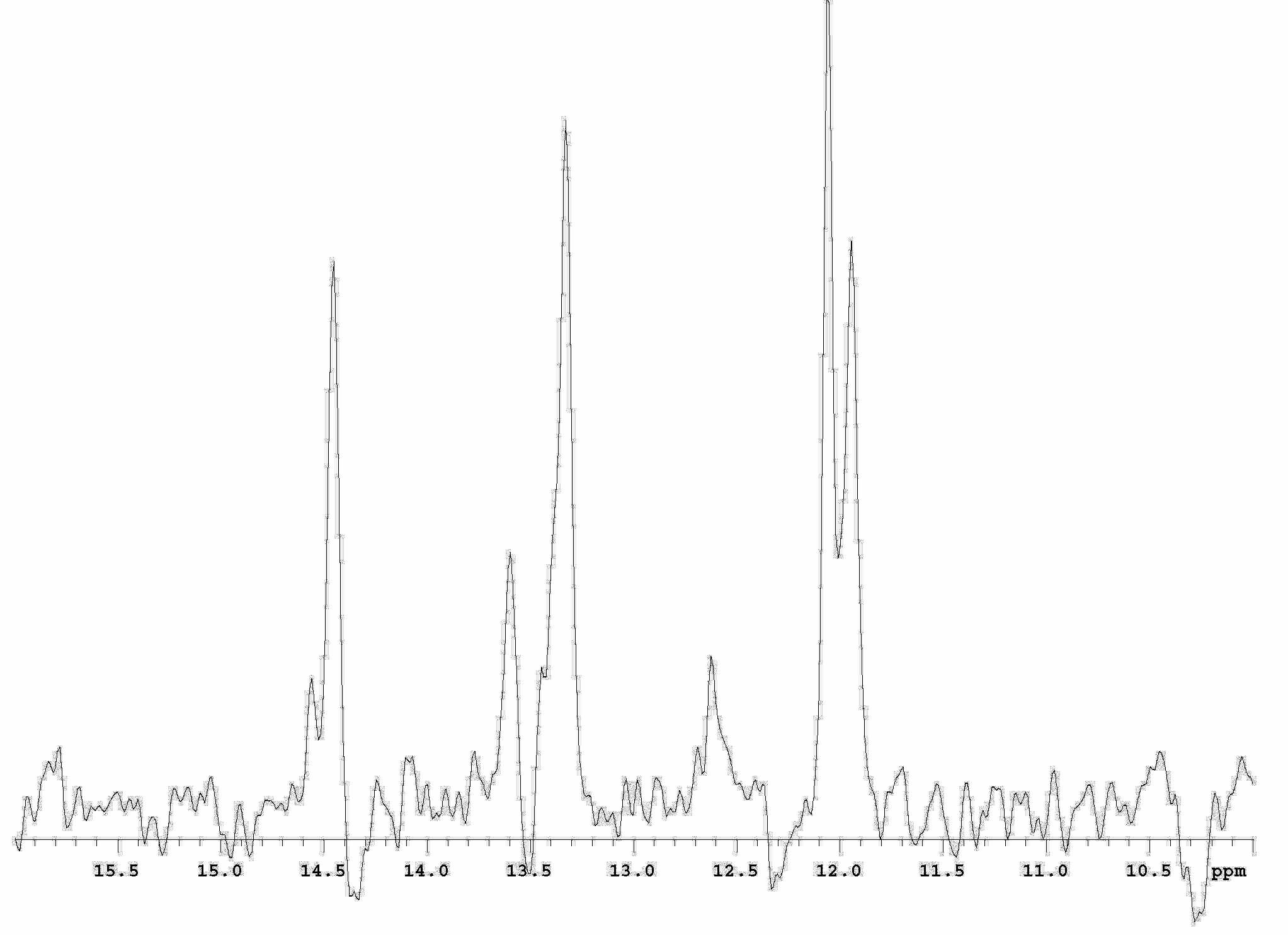

$$\begin{matrix} 5^{'}\mathrm{UGAC}\mathbf{UU}\mathrm{CUGA} \\ 3^{'}\mathrm{ACUG}\mathbf{CCCC}\mathrm{GACU} \end{matrix}$$

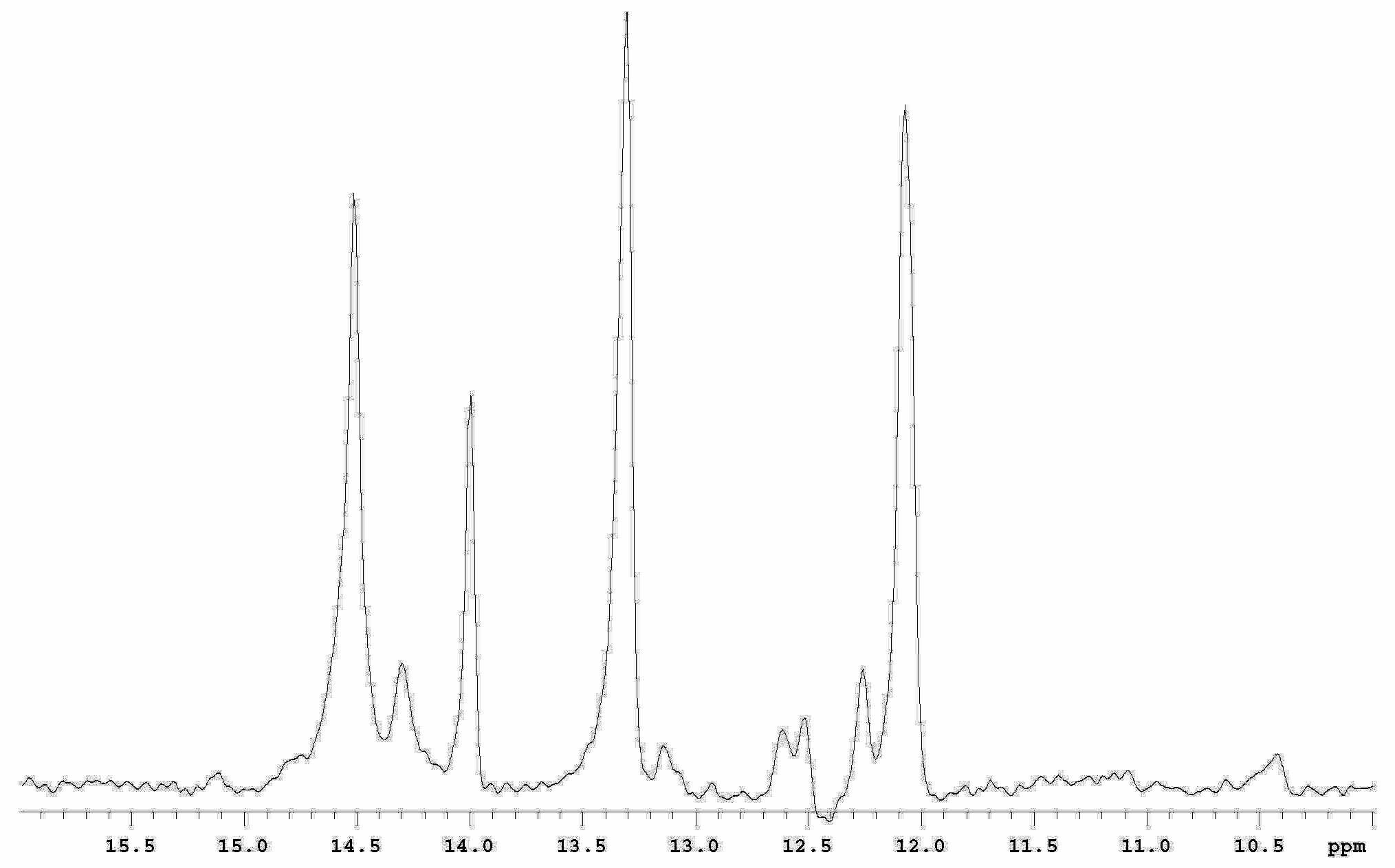

$$\begin{matrix} 5^{'}\mathrm{UGAC}\mathbf{UC}\mathrm{CUGA} \\ 3^{'}\mathrm{ACUG}\mathbf{CUCU}\mathrm{GACU} \end{matrix}$$

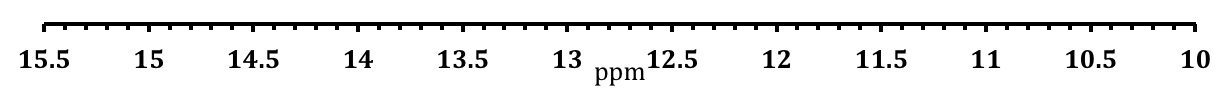


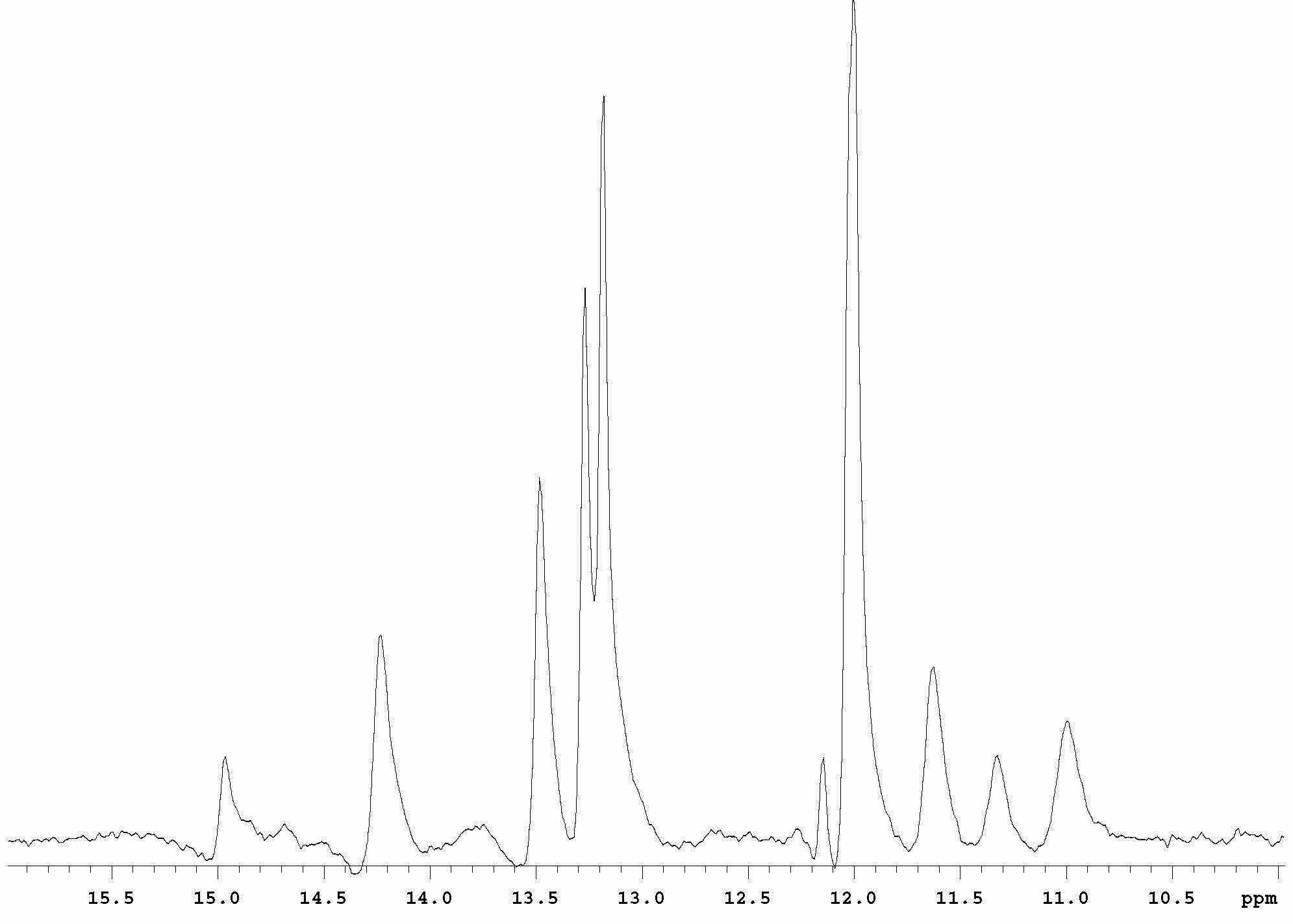

$$\begin{matrix} 5^{'}\mathrm{UGAC}\mathbf{UCUC}\mathrm{CUGA} \\ 3^{'}\mathrm{ACUG}\mathbf{CUUC}\mathrm{GACU} \end{matrix}$$

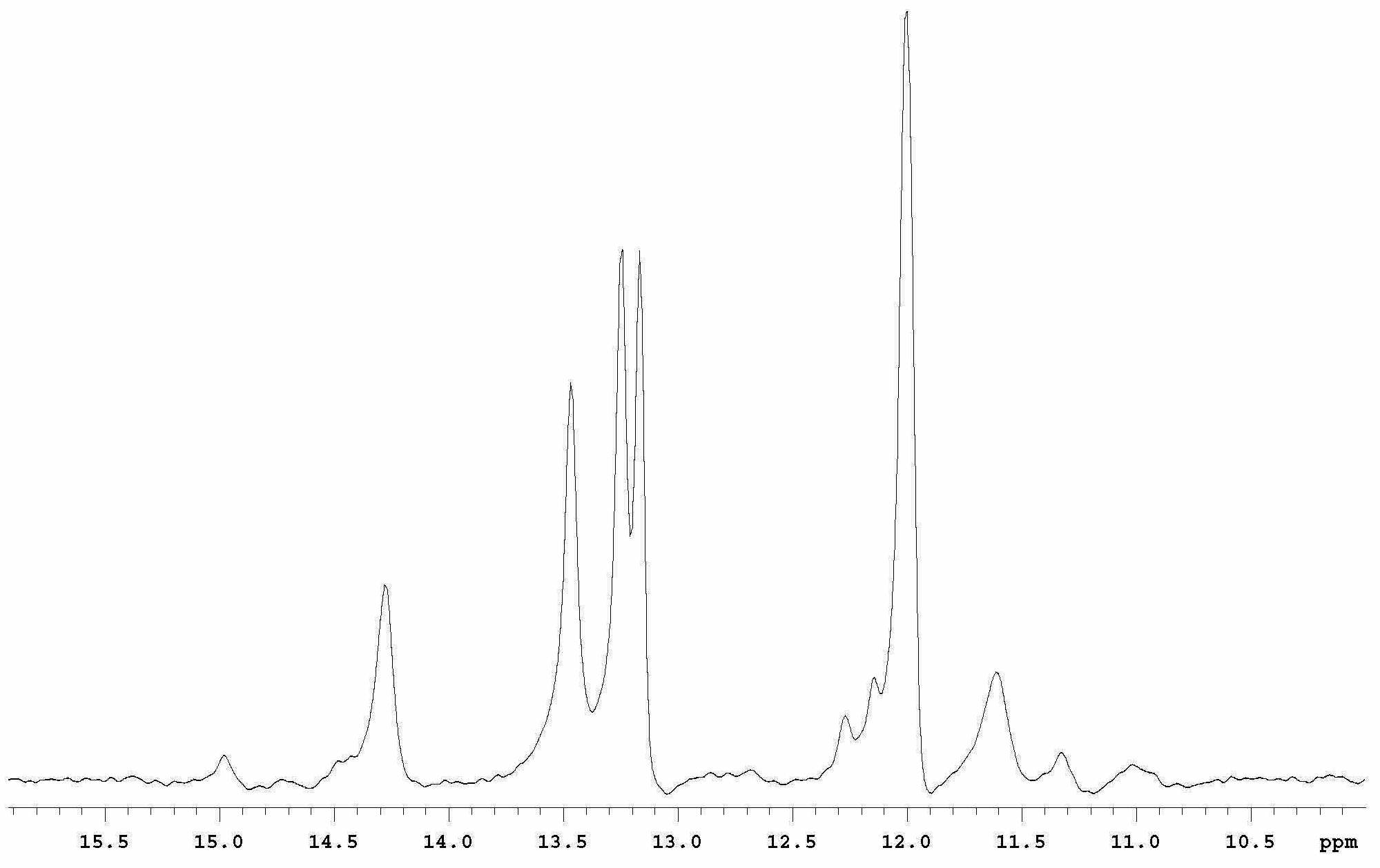

$$\begin{matrix} {\text{a}5}^{'}\mathrm{UGAC}\mathbf{UCUC}\mathrm{CUGA} \\ 3^{'}\mathrm{ACUG}\mathbf{CUUC}\mathrm{GACU} \end{matrix}$$

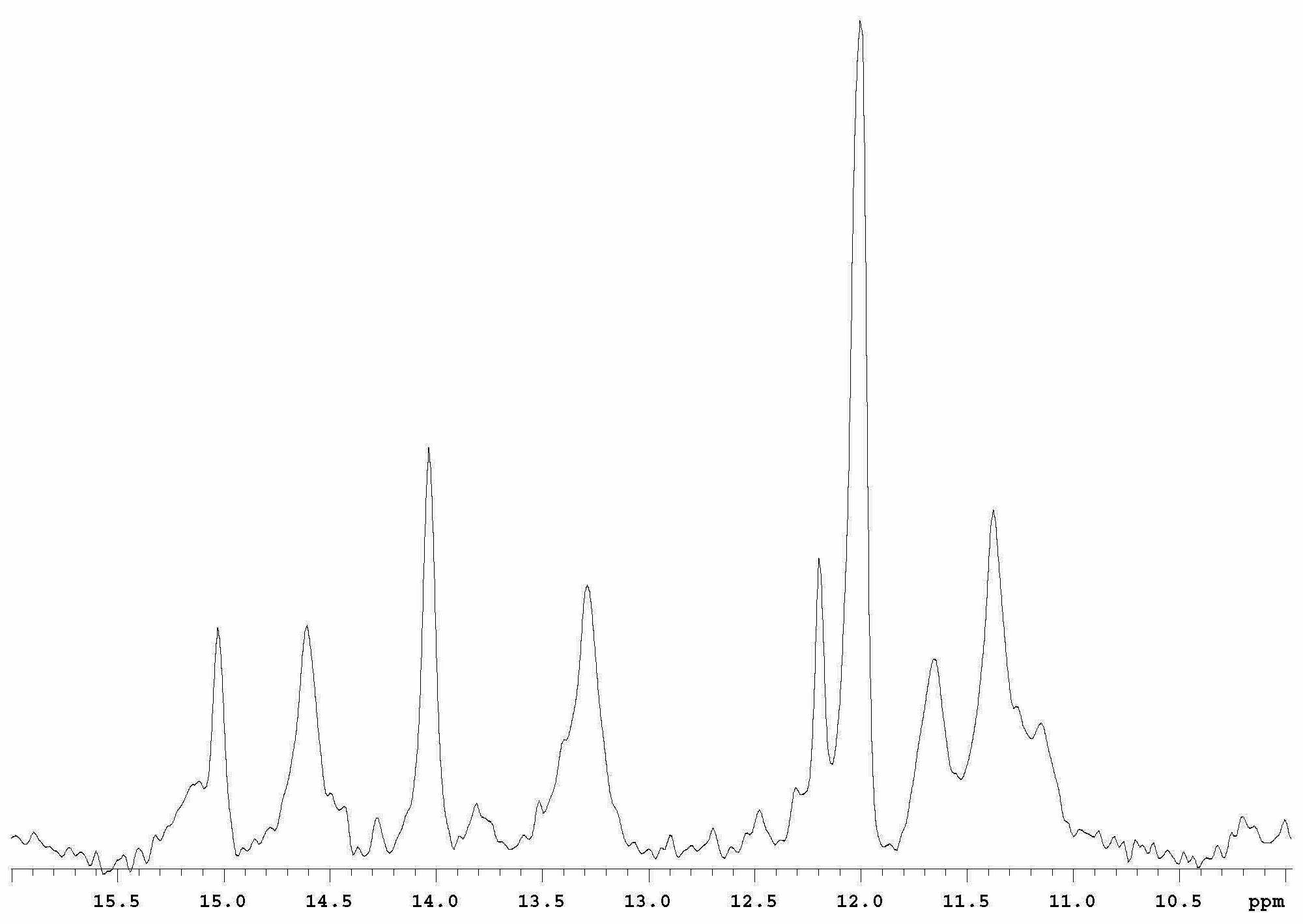

$$\begin{matrix} 5^{'}\mathrm{UGAC}\mathbf{UCUC}\mathrm{CUGA} \\ 3^{'}\mathrm{ACUG}\mathbf{CUCU}\mathrm{GACU} \end{matrix}$$

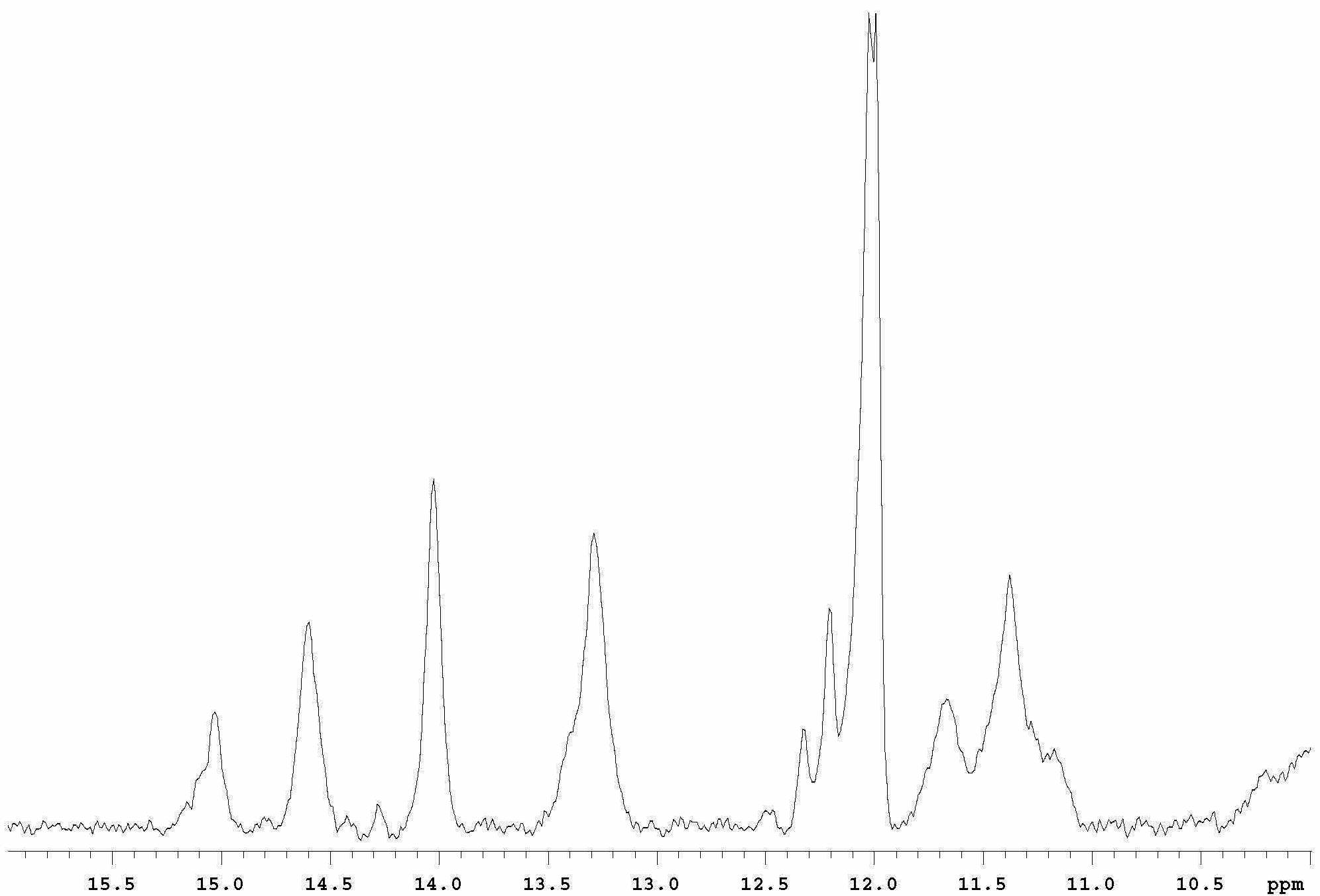

$$\begin{matrix} {\text{a}5}^{'}\mathrm{UGAC}\mathbf{UCUC}\mathrm{CUGA} \\ 3^{'}\mathrm{ACUG}\mathbf{CUCU}\mathrm{GACU} \end{matrix}$$

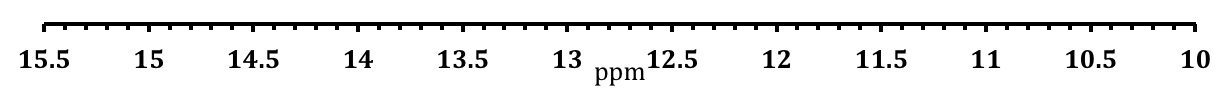


**Supporting Information Figure S1**: One-dimensional imino proton spectra at 1^o^C in 10 mM NaCl, 10 mM NaH_2_PO_4_, 0.5 mM Na_2_EDTA (pH 6) buffer. ^a^ These duplexes were analyzed at pH 7.
